# miR-615-3p targets SESN2 to inhibit neuronal mitophagy in an *in vitro* Alzheimer’s disease model by regulating the AMPK/ULK1 axis

**DOI:** 10.3389/fnmol.2026.1873619

**Published:** 2026-10-16

**Authors:** Yang Jing, DeKun Li, Li Chen, Song Li, Xiang Yuan

**Affiliations:** Department of Xiaolong Internal Medicine, Nanchong Psychosomatic Hospital, Nanchong, China

**Keywords:** Alzheimer’s disease, *in vitro* model, miR-615-3p, mitophagy, SESN2/AMPK/ULK1 axis

## Abstract

**Background:**

Alzheimer’s disease (AD) is characterized by amyloid-β accumulation, mitochondrial dysfunction, and neuronal injury. miR-615-3p is upregulated in AD, but its role in regulating mitophagy remains unclear.

**Methods:**

HT22 hippocampal neurons were treated with Aβ1-42 to establish an AD-like model. Gain-of-function and loss-of-function experiments were conducted to assess the roles of miR-615-3p and its target SESN2, which were identified through bioinformatics and dual-luciferase assays. Cell viability, apoptosis, oxidative stress, mitochondrial function, inflammatory responses, and mitophagy-related proteins were evaluated. The involvement of the AMPK/ULK1 pathway was also examined.

**Results:**

miR-615-3p overexpression significantly reduced cell viability and ATP levels while increasing apoptosis, Aβ accumulation, lactate dehydrogenase release, reactive oxygen species production, mitochondrial membrane potential loss, and inflammatory cytokine levels. Mitophagy markers, including LC3-II/LC3-I, Parkin, and PINK1, were elevated. Mechanistically, miR-615-3p downregulated SESN2 and inhibited AMPK/ULK1 phosphorylation. SESN2 overexpression reversed these effects, restoring mitochondrial function, reducing oxidative stress and inflammation, and improving cell survival.

**Conclusion:**

miR-615-3p aggravates neuronal injury by targeting SESN2 and regulating mitophagy through the AMPK/ULK1 pathway, suggesting a potential therapeutic target for Alzheimer’s disease.

## 1 Introduction

Currently, approximately 55 million people are living with dementia globally, with Alzheimer’s disease (AD) accounting for 60–70% of cases, and this number is projected to triple by 2050 as populations age (Alzheimer’s Association, 2024; Zhong et al., 2025). The pathological hallmarks of AD include extracellular amyloid-β (Aβ) plaques, intracellular neurofibrillary tangles composed of hyperphosphorylated tau protein, progressive synaptic dysfunction, and neuronal loss. Mounting evidence indicates that mitochondrial dysfunction represents a central and early event in AD pathogenesis, preceding the appearance of classical pathological lesions (Wang et al., 2020). Mitochondria are critical for meeting the high energy demands of neurons and maintaining calcium homeostasis; impairment of them leads to oxidative stress, ATP depletion, and ultimately neuronal death. Notably, defective mitochondrial quality control, particularly impaired mitochondrial autophagy (mitophagy), has emerged as a crucial contributor to AD progression (Skawratananond et al., 2025; Mary et al., 2023). In AD brains, the accumulation of damaged mitochondria with compromised membrane potential and accumulated mitochondrial DNA mutations suggests a failure of mitophagic clearance (Ashleigh et al., 2023). This mitophagy failure exacerbates the pathology related to Aβ and tau through impaired energy metabolism and increased reactive oxygen species (ROS) production, establishing a vicious cycle that accelerates neurodegeneration (Angelova and Abramov, 2018). Mitophagy dysfunction may be a significant driving factor for energy metabolism imbalance and synaptic function decline in AD neurons. However, its underlying molecular mechanisms remain to be further elucidated.

MicroRNAs (miRNAs), a class of small non-coding RNAs approximately 22 nucleotides in length, have gained substantial attention in neurodegenerative diseases, including AD. By binding to the 3′-untranslated region of target messenger RNAs, miRNAs can fine-tune the expression of multiple genes involved in Aβ metabolism, tau phosphorylation, synaptic plasticity, and neuronal survival (Wei et al., 2020). Dysregulated miRNAs, including miR-140-3p, miR-122-5p, and miR-9-5p, have been identified in the brains and biofluids of AD patients (Song et al., 2024; Li and Zheng, 2023), highlighting their potential as both biomarkers and therapeutic targets. Among these, the aberrant elevation of miR-615-3p in AD patients and the Aβ25-35-induced PC12 cell model, along with its involvement in regulating neuronal apoptosis and oxidative stress responses, has been highlighted (Liu et al., 2022). Jalaiei et al. (2025) used microarray (GSE106241, GSE157239) and reverse transcription polymerase chain reaction (RT-PCR) analyses to validate a link between miR-615-3p dysregulation, impaired synaptic function, and increased neuroinflammation in AD. miR-615-3p has emerged as one of the five key miRNA markers for AD (miR-26b-5p, miR-615-3p, miR-4722-5p, miR-23a-3p, and miR-27b-3p). The average detection accuracy is more than 84%, and it is one of the common miRNAs found in both AD and Parkinson’s disease (Zotarelli-Filho et al., 2023). However, the precise molecular mechanisms through which miR-615-3p contributes to AD pathogenesis remain incompletely elucidated.

Sestrin2 (SESN2, also known as Hi95), a stress-inducible protein belonging to the sestrin family, has emerged as a crucial regulator of cellular metabolism, oxidative stress, and autophagy (Chuang et al., 2015). Mutations in the *presenilin* genes are the main cause of familial AD; it has been reported that cells deficient in presenilin have lower expression and mTOR dysregulation, revealing a crucial role of sestrin2 in AD-associated conditions (Reddy et al., 2016). Rai et al. (2016) compared sestrin2 levels in the serum of AD patients, those with mild cognitive impairment, and control groups and found that AD patients had significantly higher sestrin2 levels than other groups. In primary rat cortical neurons, Aβ exposure increased the expression of SESN2 and LC3B-II, and SESN2 siRNA treatment could abolish LC3B-II levels and aggravate neuronal death; in addition, the autophagosome fusion inhibitor Baflomycin A1 could enhance the neurotoxicity of Aβ, which proved that SESN2 functions positively by promoting a complete autophagy process (Cordani et al., 2019). Recently, Tugce et al. concluded that phosphodiesterase inhibitors exhibited neuroprotective effects by decreasing SESN2 and modulating autophagy through the AMPK/mTOR pathway in an *in vitro* AD model (Faikoglu et al., 2025). Given the above findings, SESN2 may represent a critical node in AD-associated mitochondrial quality control failure. Previous studies have demonstrated that SESN2 can promote mitophagy by activating the AMP-activated protein kinase (AMPK) signaling pathway. The AMPK/Unc-51-like autophagy-activating kinase-1 (ULK1) axis represents a canonical signaling cascade central to the initiation and execution of autophagy, including selective mitophagy (Iorio et al., 2021). Upon activation, AMPK phosphorylates and activates ULK1, which is the mammalian homolog of yeast Atg1, thereby triggering the autophagic machinery (Song et al., 2023). Accumulating evidence suggests that SESN2-mediated AMPK/ULK1 signaling is essential for maintaining mitochondrial quality control under metabolic stress conditions.

The present study was designed to systematically investigate the miR-615-3p/SESN2/AMPK/ULK1 axis in AD pathogenesis. We propose that miR-615-3p may directly target SESN2, thereby suppressing the AMPK/ULK1-mediated mitophagy pathway and contributing to mitochondrial dysfunction in AD. Through molecular biology techniques and AD cellular models, we aimed to elucidate: (1) the regulatory effect of miR-615-3p overexpression or inhibition on neuronal mitophagy; (2) whether miR-615-3p negatively regulates SESN2 expression to modulate AMPK/ULK1 signaling; (3) manipulating SESN2 can ameliorate mitochondrial dysfunction. The findings are expected to reveal a novel regulatory node in mitochondrial quality control disruption in AD and provide a theoretical foundation for developing miRNA-based therapeutic strategies for this neurodegenerative disorder.

## 2 Materials and methods

### 2.1 Establishment and treatment of the AD cell model

The mouse hippocampal neuronal cell line HT22 (obtained from the American Type Culture Collection) was maintained in Dulbecco’s modified Eagle medium (DMEM), supplemented with 10% fetal bovine serum (FBS), 100 μg/mL streptomycin, and 100 units/mL penicillin. The cells were cultured at 37 °C, 5% CO_2_ in a humidified incubator. In addition to the control group, HT22 cells were incubated in complete medium containing 10 μM Aβ1-42 for 24 h to simulate an AD cell model. For autophagic flux analysis, the cells were treated with bafilomycin A1 (BafA1, 100 nM, 4 h; MedChemExpress, HY-100558) for 4 h before harvesting, and the protein levels of LC3 and p62 were compared between the groups with and without BafA1 treatment.

miR-615-3p mimic, mimic-negative control (NC), miR-615-3p inhibitor, inhibitor-NC, SESN2 overexpression plasmid, empty plasmid, siRNA targeting SESN2 (GCAUCAGAUACGAUGACUATT), and control siRNA were transfected into Aβ1-42-stimulated HT22 cells to harvest miR-615-3p-OE, miR-615-3p-OE-NC, miR-615-3p-KD, miR-615-3p-KD-NC, SESN2-OE, SESN2-OE-NC, SESN2-KD, and SESN2-KD-NC groups separately by utilizing Lipofectamine 3000 (Thermo Fisher Scientific) for 4–6 h. The transfection efficiency was assessed by qPCR.

### 2.2 Cell proliferation and apoptosis assays

According to the manufacturer’s protocol, Cell Counting Kit-8 solution (Biosharp, BS350B) was used to analyze the cell proliferation abilities at an absorbance of 450 nm via a microplate reader (BioTek, EL808) after incubation in a dark environment at 37 °C for 2 h.

Annexin V-FITC apoptosis detection kit (Solarbio, CA1020) was used to evaluate the apoptosis of HT22 cells by incubating with annexin V and PI dyes at room temperature for 15 min, and the cells were analyzed by flow cytometry (CytoFLEX, Beckman Coulter). Data were analyzed using FlowJo software (Becton, Dickinson and Company).

### 2.3 Immunofluorescence (ICC) staining

HT22 cells were fixed with 4% paraformaldehyde for 15 min, permeabilized with 0.1% Triton X-100, and blocked with 5% bovine serum albumin (BSA). The cells were then incubated overnight at 4 °C with an anti-SESN2 primary antibody (Proteintech, 10795-1-AP), followed by incubation with a FITC-conjugated secondary antibody for 1 h at room temperature. The nuclei were counterstained with DAPI, and the images were captured under a fluorescence microscope. The relative fluorescence intensity of SESN2 was quantified using ImageJ software.

### 2.4 Aβ1-42, lactate dehydrogenase (LDH), adenosine triphosphate (ATP), IL-1β, and IL-18 contents

Using the Aβ1–42 detection kit (Ruixin Bio, RX-Aβ1-42-H), LDH assay kit (Promega, G1780), ATP assay kit (Beyotime, S0026), IL-1β ELISA kit (mlbio, ml058059), and IL-18 ELISA kit (mlbio, ml058055), the supernatants were measured in different treatment groups.

### 2.5 Measurement of ROS

The collected HT22 cells were incubated with 10 μM DCFH-DA-A (Beyotime, S0033S) for 20 min at 37 °C or 5 μM MitoSOX Red (Beyotime, S0061S) for 15 min at 37 °C in the dark after being washed three times with 1X phosphate-buffered saline (PBS). They were then detached with trypsin/EDTA. The relative fluorescence levels of ROS were counted by flow cytometry (CytoFLEX, Beckman Coulter) and analyzed using FlowJo software (Becton, Dickinson and Company).

### 2.6 5′,6,6′-Tetrachloro-1,1′,3,3′-tetraethyl-benzimidazolyl carbocyanine iodide (JC-1) assay to detect the mitochondrial membrane potential (MMP)

HT22 cells co-administered with 2 μM JC-1 staining buffer (Beyotime, C2003S) were incubated at 37 °C and 5% CO_2_ for 30 min. After being washed with PBS and resuspended in 1 mL of JC-1 staining buffer, JC-1 aggregates were further analyzed by flow cytometry (CytoFLEX, Beckman Coulter).

### 2.7 Quantitative real-time PCR (RT-qPCR)

In HT22 cells, total RNA was extracted using TRIzol^®^ reagent and converted to synthesize cDNA using the cDNA Synthesis kit (Thermo Fisher Scientific). Then, RT-qPCR reactions were performed using a StepOnePlus Real-Time PCR system (Thermo Fisher Scientific). The relative mRNA expression levels of miR-615-3p, SESN2, AMPK, ULK1, and mitochondrial DNA were calculated using 2^−ΔΔCq^, with U6 as the normalization control.

The primers were listed as follows:

miR-615-3p:

Forward, 5′-CCCAAGCTTGGGCATAATTGGATCATAGGAAC-3′.

Reverse, 5′-CCGGAA TTCCGGGTGAATAGCTTGCAGCGTTC-3′;

SESN2:

Forward, 5′-CCTCTGGGCGAGTAGACAAC-3′.

Reverse, 5′-GGAGCCTACCAGGTAAGAACA-3′;

AMPK:

Forward, 5′--GTCAAAGCCGACCCAATGATA-3′.

Reverse, 5′--CGTACACGCAAATAATAGGGGTT-3′;

ULK1:

Forward, 5′-ACATCCGAGTCAAGATTGCTG-3′.

Reverse, 5′-GCTGGGACATAATGACCTCAGG-3′;

mitochondrial DNA:

Forward, 5′-ATCATTGGACAAGTAGCATC-3′.

Reverse, 5′-GGTGAACTCACTGGAACGGG-3′;

U6:

Forward, 5′-AAGGATGACACGCAAATTC-3′.

Reverse, 5′-GAGCAGGGTCCGAGGT-3′.

### 2.8 Western blotting

We utilized RIPA buffer (0.1 mg/mL phenylmethylsulfonyl fluoride) to lyse HT22 cells, which were then centrifuged at 13,000 × g, 4 °C for 10 min. The collected supernatant was used to quantify protein using a commercial BCA assay kit (Beyotime Biotech, P0010S). Subsequently, 20 μg protein was subjected to 10% SDS-PAGE (Epizyme Biotech), transferred onto a PVDF membrane (Millipore) for 1 h, and blocked with 5% BSA at room temperature lasting for 1 h. Then, the membranes were incubated with the primary antibodies, including anti-SESN2 (Proteintech, 10795-1-AP), anti-AMPK (Cell Signaling Technology, 5831), anti-p-AMPK (phospho-AMPKα Thr172; Cell Signaling Technology, 2535), anti-ULK1 (Sigma Aldrich, A7481), anti-p-ULK1 (phospho-ULK1 Ser555; Cell Signaling Technology, 14202S), anti-LC3II/LC3I (Abcam, ab192890), anti-Parkin (Abcam, ab77924), anti-PINK1 (Abcam, ab216144), anti-P62 (Abcam, ab109012), and anti-GAPDH (Abcam, ab9485) at 4 °C overnight. After washing with TBS-T three times, the secondary antibody (LI-COR, Inc.) was incubated at room temperature for 1 h. The ECL luminescent solution (Biosharp) was used to detect the bands of target proteins. Finally, we used ImageJ software to evaluate protein intensity and normalize it to a loading control (GAPDH).

### 2.9 Bioinformatics prediction of the miR-615-3p target

The potential target genes of miR-615-3p were predicted using the TargetScan (release 8.0, https://www.targetscan.org) and miRDB (version 6.0, https://mirdb.org) databases. The conserved binding site of miR-615-3p within the 3’-UTR of SESN2 mRNA predicted by both databases was used to design wild-type and mutant SESN2 3′-UTR sequences for the dual-luciferase reporter assay.

### 2.10 Dual-luciferase reporter assay

SESN2 mRNA 3′UTR gene fragment (SESN2 Wt) and mutant fragment (SESN2 Mut) combined with miR-615-3p were synthesized and inserted into the pmirGLO vector. After restriction enzyme digestion, T4 DNA ligase was used to insert the target fragment into the vector, and Lipofectamine 3000 reagent was used to co-transfect 300 ng plasmid with 50 nM NC mimic or miR-132 mimic into HEK293T cells. Then, the cells were incubated at 37 °C in a humidified incubator containing 5% CO_2_ for 48 h, after which they were collected and lysed. The Dual-Luciferase^®^ Reporter kit was used to measure luciferase activity.

### 2.11 Statistical analysis

Data, expressed as means ± standard error, were repeated ≥ three times independently and analyzed using GraphPad Prism software (version 6.01). The comparison of groups was carried out using one-way ANOVA followed by Tukey’s *post-hoc* test. Statistical significance was defined as *p < 0.05, **p < 0.01, and ***p < 0.001.

## 3 Results

### 3.1 Effect of miR-615-3p on proliferation, apoptosis, and mitochondrial autophagy on Aβ1-42-stimulated HT22 cells

The study utilized HT22 cells to create an AD cell model in order to examine the effects of miR-615-3p in adipose tissue on cell proliferation, apoptosis, and mitochondrial autophagy. Firstly, RT-qPCR was used to validate the relative expression of miR-615-3p in both HT22 cells (Figure 1A) and Aβ_1-42_-treatd HT22 cells (Figure 1B). miR-615-3p and Aβ1-42 were significantly elevated in the model group; the use of the miR-615-3p mimic significantly reduced cell viability and promoted cell apoptosis and Aβ1-42 expression. These effects could be counteracted by the miR-615-3p inhibitor (Figures 1C–E). Then, mitochondrial function changes (intracellular ROS and LDH), MMP, and autophagy-related proteins (including LC3II/LC3I, Parkin, PINK1, P62) were also investigated (Figures 1F–L). In the model group, LDH, ROS fluorescence intensity, mitochondrial DNA D-loop and autophagy-related proteins were significantly higher; however, mitochondrial membrane potential and ATP levels were significantly lower than in the control group, and miR-615-3p overexpression enhanced the above altered tendency, which was reversed by the miR-615-3p inhibition (all *p* < 0.05). To further determine whether the increased LC3-II and p62 levels reflect enhanced autophagosome formation or blocked autophagic degradation, we performed an autophagic flux assay using the lysosomal inhibitor bafilomycin A1 (BafA1) according to following the established guidelines. In the control and model groups, BafA1 treatment significantly further increased LC3-II and p62 levels, whereas in the miR-615-3p-OE group, BafA1 failed to further accumulate LC3-II and p62 (all *p* > 0.05, Supplementary Figure S1). These results indicate that autophagic flux is attenuated or blocked in miR-615-3p-overexpressing cells, supporting that the accumulation of autophagy-related proteins results from impaired autophagosome–lysosome degradation rather than increased autophagosome formation alone. In addition, the inflammatory factors IL-1β and IL-18 levels, as shown by ELISA analysis, were significantly upregulated in the model group compared to the control group, and the extra use of miR-615-3p mimic could aggravate their expressions, but the suppression of miR-615-3p would reduce IL-1β and IL-18 levels (Figure 1M, all *p* < 0.001).

**Figure 1:**
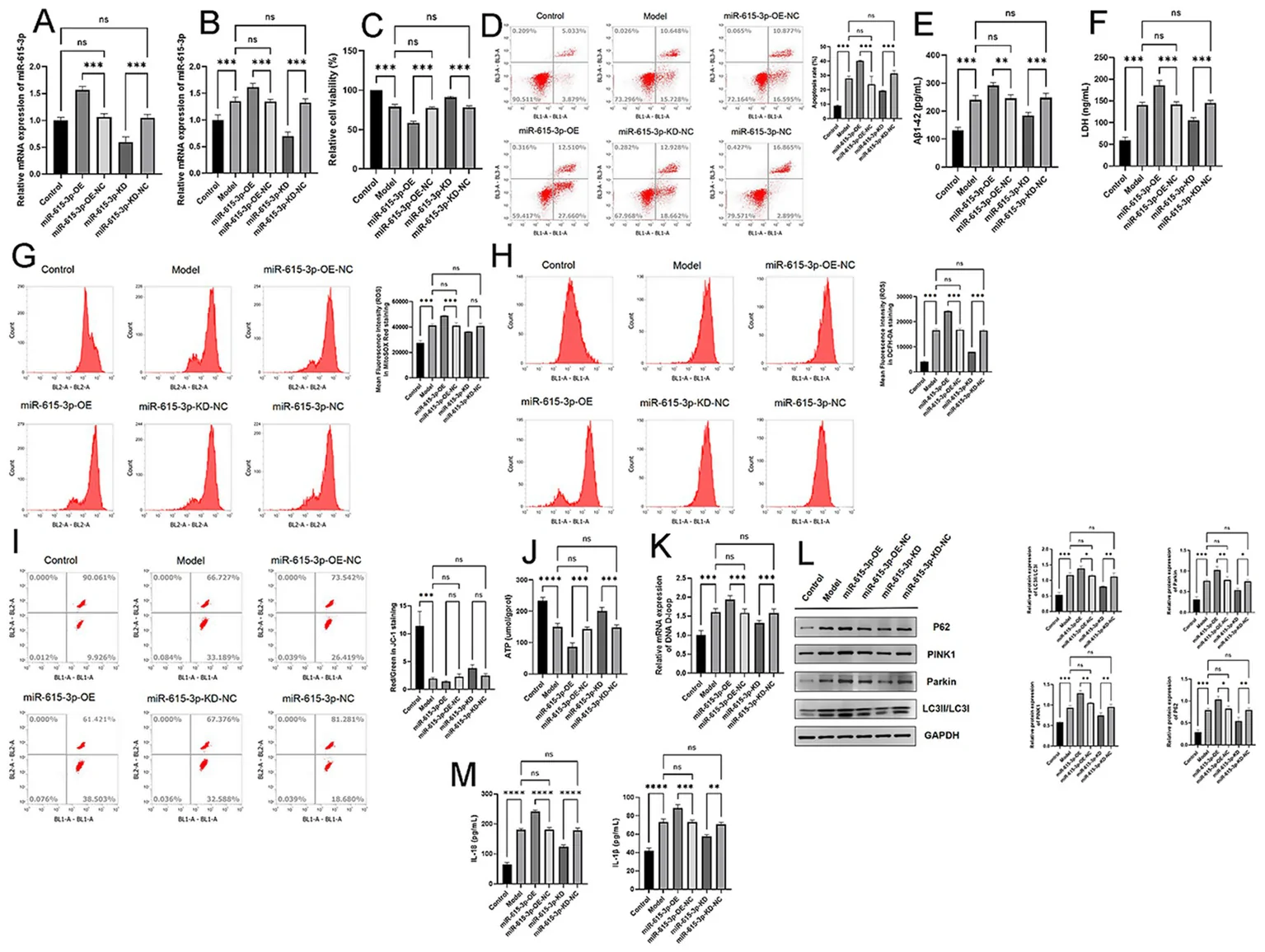
The proliferation, apoptosis, mitophagy, and inflammation changes in Aβ1-42-stimulated HT22 cells after miR-615-3p mimic or inhibitor treatments. **(A,B)** The relative miR-615-3p mRNA levels in HT22 cells and Aβ1-42-stimulated HT22 cells. **(C)** The cell viability through CCK8 assay. **(D)** The apoptosis abilities were assessed through Annexin V-FITC assay. **(E,F)** ELISA analyzed the Aβ1-42, lactate dehydrogenase (LDH) production in cells. **(G,H)** Intracellular reactive oxygen species (ROS) were determined by DCFH-DA-A and MitoSOX Red staining, respectively. **(I)** JC-1 staining measured the mitochondrial membrane potential (MMP). **(J)** The adenosine triphosphate (ATP) production in cells was quantified. **(K)** qRT-PCR analyzed the relative mitochondrial DNA expression. **(L)** Western blotting detected the LC3II/LC3I, Parkin, PINK1, and P62 proteins. **(M)** ELISA assay was to measure the inflammatory factors (IL-1β, IL-18).

### 3.2 miR-615-3p downregulates SESN2 to regulate AMPK/ULK1 axis

The potential binding site of miR-615-3p in the conserved 3’-UTR region of SESN2 mRNA was predicted using the TargetScan and miRDB databases (Figure 2A), suggesting that miR-615-3p may inhibit the translation of SESN2 and promote its mRNA degradation. The dual-luciferase reporter assay in Figure 2B demonstrated the interaction between miR-615-3p and SESN2, as co-transfection of miR-615-3p mimics with SESN2-WT significantly reduced the relative luciferase activity (*p* < 0.001). Conversely, the SESN2-WT and miR-615-3p mimic constructs showed no changes in luciferase activity. This indicates that the mutation or deletion of the miR-615-3p binding site was capable of abrogating the regulatory effect of miR-615-3p on SESN2 expression. Next, it was observed that the relative SESN2 mRNA and protein expressions were significantly decreased in the model group, and the overexpression of miR-615-3p would downregulate the SESN2 levels (Figures 2C,D). Considering that SESN2 has been found to be a key regulatory factor in the AMPK/ULK1 pathway, the regulatory effect of miR-615-3p overexpression or inhibition on the AMPK/ULK1 axis was explored. It showed that the application of miR-615-3p mimic significantly reduced the levels of p-AMPK (Thr172) and p-ULK1 (Ser555), similar to the results caused by co-transfection with SESN2 siRNA; however, miR-615-3p inhibitor or SESN2 overexpression would significantly increase the p-AMPK and p-ULK1 proteins (all *p* < 0.05, Figures 2D,F).

**Figure 2:**
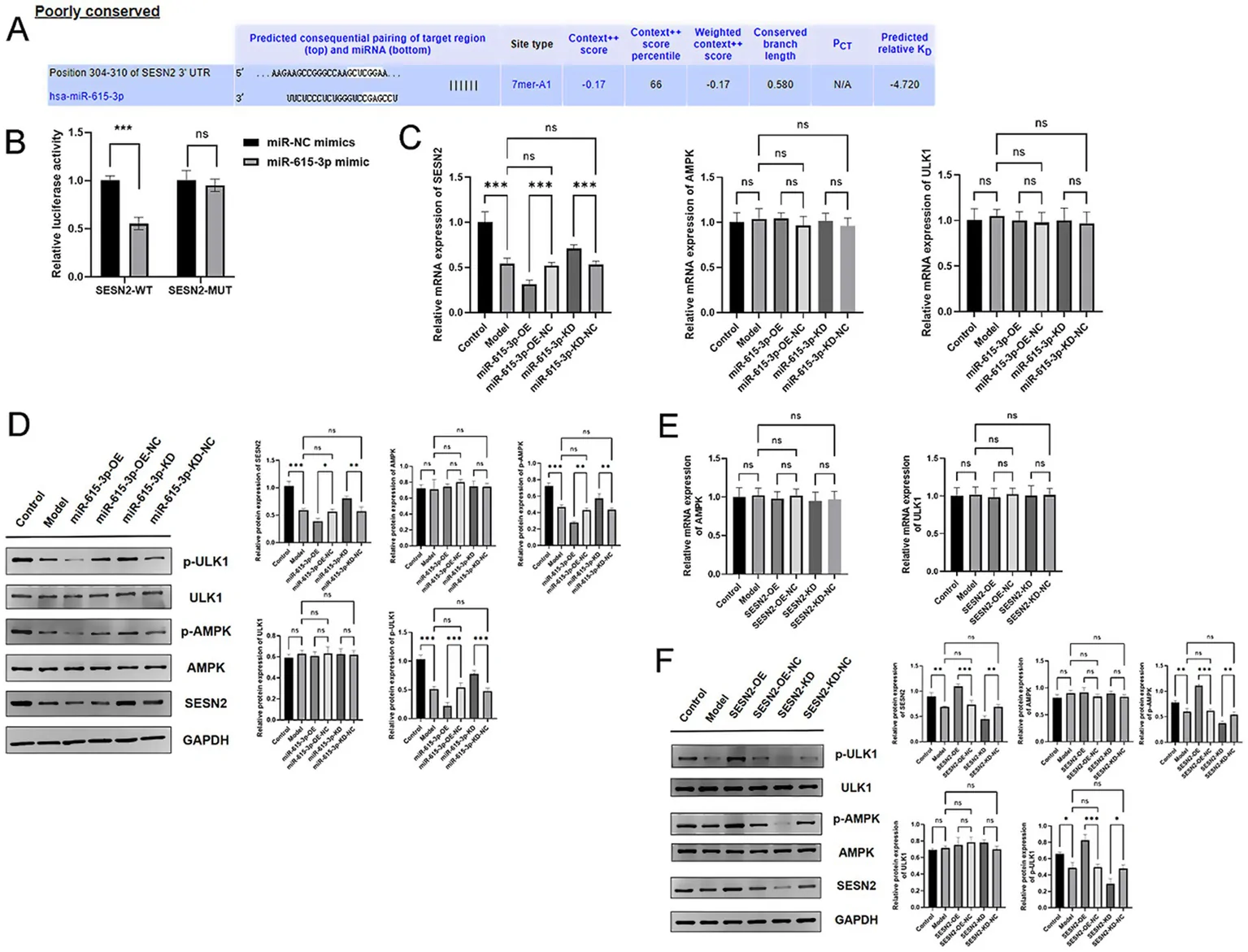
miR-615-3p downregulates SESN2 to regulate the AMPK/ULK1 axis. **(A,B)** Bioinformatics analysis integrated with dual-luciferase reporter assay was used to identify SESN2 as a potential target of miR-615-3p. **(C,D)** qRT-PCR and western blotting were used to analyze the relative miR-615-3p, SESN2, AMPK, p-AMPK, ULK1, and p-ULK1 expressions in Aβ1-42-stimulated HT22 cells after miR-615-3p mimic or inhibitor treatments. **(E,F)** qRT-PCR and western blotting were used to analyze the relative miR-615-3p, SESN2, AMPK, p-AMPK, ULK1, and p-ULK1 expressions in Aβ1-42-stimulated HT22 cells after SESN2 overexpression or knockout treatments.

### 3.3 The overexpression of SESN2 plays an inhibitory role in mitochondrial autophagy of Aβ1-42-stimulated HT22 cells

SESN2-OE and SESN2 siRNA plasmids were constructed and verified for efficiency in this study (Figures 3A,B). Consistently, immunofluorescence staining showed that the SESN2 signal was significantly increased in the SESN2-OE group and markedly decreased in the SESN2-KD group compared with the corresponding NC groups (*p* < 0.01 or *p* < 0.001, Supplementary Figure S2). SESN2 was significantly decreased in the model group than in the control group, and the SESN2-OE transfection would induce cell proliferation and suppress cell apoptosis and Aβ1-42 expression; these changes were significantly reversed by transfection with SESN2 siRNA (all *p* < 0.01, Figures 3C–E). The subsequent evaluation of mitochondrial autophagy (Figures 3F–L) indicated that the overexpression of SESN2 could reduce LDH, ROS fluorescence intensity, mitochondrial DNA D-loop, LC3II/LC3I, Parkin, PINK1, and P62 levels but increase mitochondrial membrane potential and ATP levels; the use of SESN2 siRNA exerted a contrary effect (all *p* < 0.05). The ELISA analysis in Figure 3M showed that the increased levels of IL-1β and IL-18 in model cells were significantly enhanced by SESN2 knockout but significantly suppressed by overexpressing SESN2 (all *p* < 0.01).

**Figure 3:**
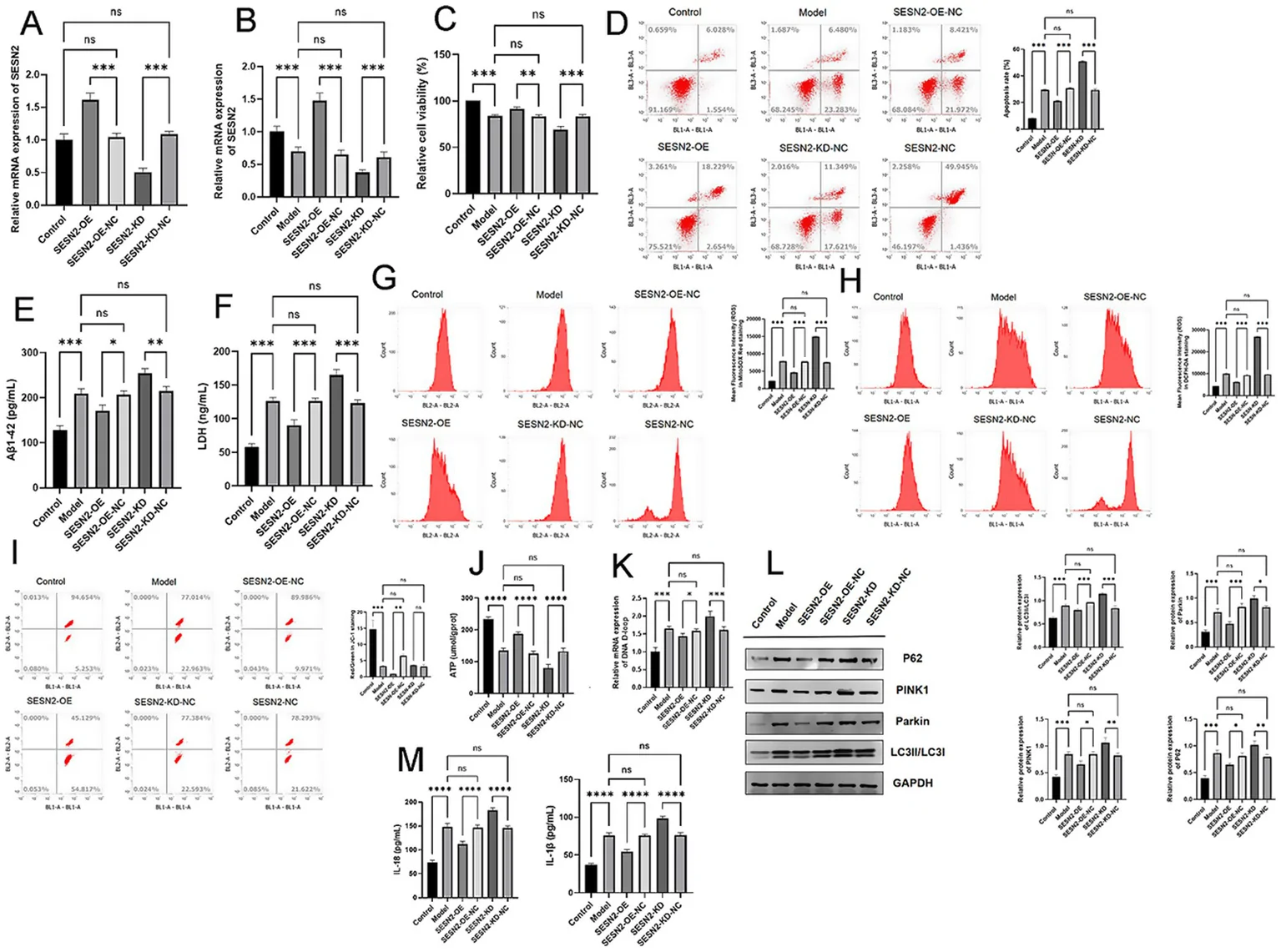
The effect of SESN2 overexpression or knockout on proliferation, apoptosis, mitophagy, and inflammation changes in Aβ1-42-stimulated HT22 cells. **(A,B)** The relative SESN2 mRNA levels in HT22 cells and Aβ1-42-stimulated HT22 cells. **(C)** The cell viabilities were evaluated through CCK8 assay. **(D)** The apoptosis abilities through Annexin V-FITC assay. **(E,F)** The Aβ1-42-stimulated lactate dehydrogenase (LDH) production was measured. **(G,H)** DCFH-DA-A and MitoSOX Red staining were used to detect the fluorescence intensity of intracellular reactive oxygen species (ROS). **(I)** JC-1 staining analysis of the mitochondrial membrane potential (MMP). **(J)** The quantification of adenosine triphosphate (ATP) production via ELISA analysis. **(K)** qRT-PCR analyzed the relative mitochondrial DNA expression. **(L)** Western blotting detected the LC3II/LC3I, Parkin, PINK1, and P62 proteins. **(M)** The inflammatory factors (IL-1β, IL-18) in cells were measured.

## 4 Discussion

AD is characterized by progressive cognitive dysfunction, and its pathogenesis involves neuronal apoptosis, mitochondrial dysfunction, inflammatory reaction, and other pathological processes. This research revealed a new mechanism in which miR-615-3p inhibits neuronal mitochondrial autophagy by targeting SESN2 to regulate the AMPK/ULK1 axis in the HT22 cell model treated with Aβ1-42, which provided a new perspective for understanding the molecular pathological mechanism of AD.

### 4.1 Regulation of miR-615-3p in neurodegenerative diseases

miRNAs play a key regulatory role in the occurrence and development of AD. Recent studies have shown that miR-615-3p may be involved in the pathogenesis of AD by regulating FoxO and ErbB signaling pathways (Liu et al., 2022). In this study, it was found that under Aβ1-42-induced neurotoxicity, the overexpression of miR-615-3p significantly inhibited cell proliferation and ATP production, and promoted cell apoptosis, Aβ accumulation, LDH release, ROS production, and MMP deterioration (Figure 1). Therefore, miR-615-3p critically promotes the pathological process of AD, which differs from the previously reported role of miR-615-3p in promoting the proliferation and migration in gastric cancer (Wang et al., 2018), indicating that miRNAs may exhibit tissue-specific functional differences across cell types and pathological backgrounds. It is worth noting that the inflammatory response is enhanced after overexpressing miR-615-3p, consistent with the core pathological features of neuroinflammation in AD (Lonkar et al., 2025), supporting the clinical value of miR-615-3p as a potential AD biomarker.

### 4.2 Dual regulation mechanism of mitophagy in AD

PINK1/Parkin-mediated mitophagy is the most deeply studied pathway at present in AD. Here, the use of miR-615-3p mimic led to an increase in the LC3-II/LC3-I ratio, accompanied by the upregulation of Parkin, PINK1, and P62 levels (Figure 1L), showing that mitophagy might be blocked. Accordingly, Aβ and tau proteins can interfere with the translocation of Parkin to mitochondria through various mechanisms in an AD model, resulting in mitophagy disorder and damaged mitochondrial accumulation (Goudarzi et al., 2021). The results of this study prove the complex understanding of the role of mitophagy in AD. On the one hand, enhancing mitophagy is regarded as a potential therapeutic strategy for AD. The activation of Parkin can reduce the Aβ load and improve synaptic plasticity in an AD mouse model (Xian and Liou, 2021). Exercise intervention can enhance mitophagy and reduce Aβ levels by upregulating Parkin (Goudarzi et al., 2021). On the other hand, simply upregulating the expression of mitophagy-related proteins without restoring the complete autophagy flow may lead to autophagy accumulation and increased cytotoxicity. At present, miR-615-3p overexpression aggravates cell damage, which may be explained by the fact that although the expression of mitophagy-related proteins is upregulated, the degradation function of downstream lysosomes may be impaired, resulting in the autophagy accumulation and formation of an “autophagy stress” state. Consistently, our new BafA1-based flux assay confirmed that the degradation capacity of the autophagy-lysosomal system is compromised in miR-615-3p-overexpressing neurons, in line with recent evidence that lysosomal dysfunction is a primary trigger of autophagic build-up and neuronal death in AD (Nixon, 2024; Lee et al., 2022). It has been shown that the accumulation of the C99 fragment (but not Aβ itself) is closely related to lysosomal-autophagy dysfunction, which can destroy the integrity of autophagy flow (Mary et al., 2023).

### 4.3 The core position of SEN2-AMPK-ULK1 axis in neuroprotection

The current mechanism analysis showed that miR-615-3p inhibited the levels of p-AMPK and p-ULK1 by downregulating the expression of SESN2 (Figure 2), thereby blocking the initiation of mitophagy. Specifically, the antibody used detects AMPKα phosphorylated at Thr172, the canonical activating site of AMPK, and ULK1 phosphorylated at Ser555, an AMPK-directed activating site essential for autophagy initiation (Mary et al., 2025). Therefore, the concurrent reduction of p-AMPK (Thr172) and p-ULK1 (Ser555) indicates a genuine suppression of the AMPK–ULK1 cascade rather than an inhibitory phosphorylation event. In osteosarcoma, Yu et al. (2024). discovered that miR-615-3p negatively regulated SESN2 expression to facilitate OS progression and activated the mammalian target of rapamycin (mTOR) pathway. Pan et al. (2023) observed that SESN2 promoted the expression of mitophagy receptors (BNIP3 and FUNDC1) by activating AMPK in a hypoxic nerve injury model and plays a neuroprotective role, but the deletion of SESN2 leads to the inhibitory activation of AMPK and the weakening of the mitophagy signal, which aggravates nerve injury. AMPK acts as the core regulator of cell energy metabolism to directly phosphorylate ULK1 (Ser555 site), and then starts autophagy (Pradeepkiran et al., 2022). The downregulation of p-AMPK and p-ULK1 expression observed in this study suggests that miR-615-3p inhibits the initiation of mitophagy through the SEN2/AMPK/ULK1 cascade reaction, forming a vicious circle. Notably, the overexpression of SESN2 showed the opposite protective effect to the miR-615-3p mimic, which verified the reliability of SESN2 as a functional target of miR-615-3p. SESN2 has been confirmed to not only protect neurons against oxidative stress but also influence the pathological process related to aging by regulating TORC1 activity. In a *Drosophila* model, SESN deletion accelerates age-related pathological changes, including mitochondrial dysfunction and muscle degeneration, and an AMPK activator can reverse these phenotypes (Budanov, 2011). These findings support the potential of the SESN2-AMPK axis as a therapeutic target for AD.

### 4.4 Research limitations and future prospects

Some limitations exist in this study. First, the study was conducted only in the HT22 cell line, lacking verification in primary neurons and animal models *in vivo*. It is necessary to verify the pathological effect of the miR-615-3p/SESN2/AMPK axis in APP/PS1 transgenic mice (da Rocha et al., 2025) or an Aβ injection model. In addition, as a mouse-derived immortalized hippocampal cell line, HT22 cells do not fully recapitulate the molecular features of primary or human neurons, which may limit the direct translation of our findings; therefore, future studies should validate the miR-615-3p/SESN2/AMPK/ULK1 axis in primary neurons and *in vivo* AD models to confirm its pathological and therapeutic relevance. Second, this study mainly focuses on the PINK1/Parkin-mediated mitophagy pathway and does not discuss whether PINK1/Parkin-independent pathways (such as receptor-mediated mitophagy) (Yang et al., 2025) participate in the regulatory network of miR-615-3p. Subsequent studies should comprehensively evaluate the relative contributions of different mitophagy modes in AD. Third, although we have now performed a bafilomycin A1-based autophagic flux assay and found that autophagic degradation is blocked in miR-615-3p-overexpressing cells, the functional status of lysosomes has not been directly visualized. The mRFP-GFP-LC3 double fluorescence reporting system, in which yellow puncta represent autophagosomes and red-only puncta represent autolysosomes, should be used in future studies to directly distinguish impaired autophagosome-lysosome fusion from defective lysosomal degradation. Fourth, miR-615-3p may have multiple target genes. This experiment only verified the regulatory role of SESN2. A co-transfection rescue assay should be explored and other potential targets need to be identified by RNA-seq or proteomics.

Based on the results of this study, the following research directions are as follows: (1) developing miR-615-3p antagonists or SESN2 agonists as potential therapeutic strategies for AD; (2) exploring the expression level of miR-615-3p in the cerebrospinal fluid or blood of AD patients and its diagnostic value; (3) studying the interaction between miR-615-3p and pathology and neuroinflammation of Tau; (4) evaluating the long-term effect of targeting SESN2/AMPK axis on improving the cognitive function of AD.

## 5 Conclusion

Collectively, miR-615-3p might be a negative regulator of neuronal survival and mitochondrial function in AD pathogenesis through the SESN2-dependent modulation of the AMPK/ULK1 autophagy axis. These findings provide novel insights into the molecular mechanisms underlying AD-related neurodegeneration and highlight the potential of targeting the miR-615-3p/SESN2/AMPK pathway for the development of therapeutic interventions against AD. Further *in vivo* studies are warranted to validate these observations and explore the translational implications of this regulatory network in AD treatment.

## Data Availability

The raw data supporting the conclusions of this article will be made available by the authors, without undue reservation.

## Supplementary material

SUPPLEMENTARY FIGURE S1 Autophagic flux analysis by bafilomycin A1 (BafA1) treatment. HT22 cells in the Control, Model, and miR-615-3p-OE groups were treated with or without BafA1 (100 nM, 4 h), and the protein levels of LC3 and p62 were detected by western blotting. GAPDH served as the loading control. BafA1 further increased LC3 and p62 levels in the control and model groups but not in the miR-615-3p-OE group (ns, *p* > 0.05), indicating blocked autophagic flux after miR-615-3p overexpression. *n* = 3 independent experiments.

SUPPLEMENTARY FIGURE S2 Immunofluorescence (ICC) staining of SESN2 in HT22 cells. SESN2 (green) and nuclei (DAPI, blue) were detected in the Control, SESN2-OE, SESN2-OE-NC, SESN2-KD, and SESN2-KD-NC groups. The relative fluorescence intensity of SESN2 was quantified on the right. SESN2 was significantly increased after SESN2-OE transfection (****p* < 0.001 vs. SESN2-OE-NC) and significantly decreased after SESN2-KD transfection ^(**^*p* < 0.01 vs. SESN2-KD-NC); ns, not significant. *n* = 3 independent experiments.
